# Current Approach to the Diagnosis and Treatment of Heterozygote and Homozygous FH Children and Adolescents

**DOI:** 10.1007/s11883-021-00926-3

**Published:** 2021-05-08

**Authors:** Hofit Cohen, Claudia Stefanutti, and The Mighty Medic Satellite Research Group for Pediatric Dyslipidemia, Serafina Di Giacomo, Claudia Morozzi, Kurt Widhalm, Bojko B. Bjelakovic, Andrea Berni, Francesco Martino, Giovanna Bosco

**Affiliations:** 1 The Bert W. Strassburger Lipid Center, The Chaim Sheba Medical Center, Tel-Hashomer, Ramat Gan Israel; 2 Sackler Faculty of Medicine, Tel Aviv University, Tel Aviv, Israel; 3 Department of Molecular Medicine, Lipid Clinic and Atherosclerosis Prevention Centre, Immunohematology and Transfusion Medicine, Regional Centre for Rare Diseases, Extracorporeal Therapeutic Techniques Unit – Severe Genetic Dyslipidemias, Umberto I Hospital, ‘Sapienza’ University of Rome, Rome, Italy

**Keywords:** Pediatric homozygous-heterozygous familial hypercholesterolemia, Low-density lipoprotein cholesterol, Statin, Proprotein convertase subtilisin/kexin type 9, Lomitapide, Lipoprotein apheresis

## Abstract

**Purpose of Review:**

To elucidate the current approach of care in pediatric patients with familial hypercholesterolemia (FH). We sought an answer to the question whether the advances and major changes in lipid management are relevant and apply to children and adolescents.

**Recent Findings:**

Latest research findings clearly demonstrate that lowering cholesterol levels at a young age prevents vascular atherosclerotic changes and decreases cardiovascular events in adulthood and emphasizes the importance of early detection and intervention in the pediatric FH patients group.

**Summary:**

FH is a common genetic disease caused by mutations in genes associated with the metabolism of low-density lipoproteins (LDL). The hallmark of FH is elevated LDL cholesterol (LDL-C) levels from birth and premature atherosclerotic cardiovascular disease (ASCVD). Often FH is either undiagnosed or diagnosed with a considerable delay, leading to vascular atherosclerotic changes and cardiovascular disease. Prompt identification of FH subjects is essential, to initiate early preventive measures. Safe and efficient pharmacological agents are approved for use in children and adolescents. Statins are the first line of therapy, in combination of ezetimibe. Unfortunately, these drugs do not warrant the achievement of therapeutic target, especially in HoFH patient. In the latter, lipoprotein apheresis (LA), which has been shown to be safe and effective, is strongly recommended. Finally, the new drugs still under study will allow a multimodal customized treatment. Lowering cholesterol levels at a young age hinders vascular atherosclerotic changes decreasing cardiovascular events in adulthood. Therefore, early detection, diagnosis, and intervention in FH patients are priority objectives.

## Introduction

Familial hypercholesterolemia (FH) is a common congenital metabolic disorder, characterized by substantial elevation of plasma cholesterol levels from birth and consequently premature ASCVD [1].

## Prevalence of FH

Worldwide, the prevalence of heterozygous FH (HeFH) is estimated to be between one in 200 to 300 individuals. However, it is higher in populations with a high rate of consanguinity. Homozygous familial hypercholesterolemia (HoFH) is relatively rare, with an estimated prevalence of 1:300,000 to 1:400,000 [2].

## Genetics of FH

The phenotype of FH is caused by several mutations affecting the metabolism of LDL. The most common mutation is in the gene encoding for the LDL receptor (LDLR). Goldstein and Brown demonstrated that this classic phenotype of FH results from defects in the cell surface receptor that removes LDL particles from plasma [3]. Less common sites are the genes encoding for apolipoprotein B (APOB) [4] and proprotein convertase subtilisin/kexin 9 (PCSK9) [5]. Specifically, the distribution of the mutations among the patients with one out of the above-mentioned mutations is 85–90% for the LDLR mutations, 2–4% are due to gain-of-function PCSK9 mutations, and 1–12% are due to APOB mutation [6]. FH is an autosomal dominant disorder in most cases; hence, FH homozygotes are more severely affected than heterozygotes [7].

## Diagnosis of Heterozygous FH

FH can be diagnosed on either by phenotypic criteria or by a genetic diagnosis. Phenotypic diagnosis relies on biochemical and clinical criteria, i.e., elevated LDL-C levels and a positive family history of elevated LDL-C and premature coronary heart disease (CHD). Confirmed diagnosis may be achieved by genetic analysis [8]. The characteristic physical finding of tendon xanthomata and xanthelasmas is infrequently seen in HeFH children and is usually present in HoFH children.

Three sets of phenotype-associated criteria for FH exist, in which clinical diagnostic tools are used for the identification of FH: the United States Make Early Diagnosis to Prevent Early Death (MedPed) Program, the Simon Broome Register Group in the UK, and the Dutch Lipid Clinic Network. In the Simon Broome Register Group, the criteria for FH comprise of plasma cholesterol levels, clinical characteristics, molecular diagnosis, and family history of ASCVD [9]. The MedPed criteria use cut points for plasma total cholesterol (TC) levels specific to an individual’s age and family history [10]. The Dutch Lipid Clinic Network criteria are like the Simon Broome Register criteria [11]. Points are assigned for family history of hyperlipidemia or premature coronary and/or vascular disease, clinical characteristics of tendon xanthomata, elevated LDL-C, and/or functional mutation of the LDLR, APOB, or PCSK9 genes.

## Genetic Diagnosis

While genetic confirmation of FH, by demonstrating of one of the causative mutations, is encouraged by several research groups and organizations, there is no universal agreement for the indication of mandatory genetic testing once the phenotype of FH is found. The advantages of genetic testing are effective identification of all affected family members by means of cascade screening of relatives and by attainment of a definite diagnosis. Genetic testing increases the compliance for life-long preventive therapy; additionally, genetic counseling and prenatal diagnosis can be offered to all family members [12]. Nevertheless, genetic testing is not universally available and is costly; moreover, FH can be caused by an accumulation of LDL-C-raising alleles. This is confirmed by reports that more than half of patients are mutation negative. Consequently, a substantial number of clinically diagnosed patients with FH but without a known mutation could possibly be polygenic cause [13].

## Cascade Screening—Testing of Families

Cascade Screening occurs when screening of one patient results in a diagnosis that requires screening of additional family members for that same diagnosis. Following the diagnosis of FH at the proband, it is essential to examine additional family members for FH [14]. Current recommendations suggest that screening for FH should be completed in first- and second-degree relatives of the patient. Cascade screening either can be achieved by lipid panel testing or targeted genetic testing when molecular diagnosis has been made [2, 15].

## Management of Heterozygous FH

**Fig. 1:**
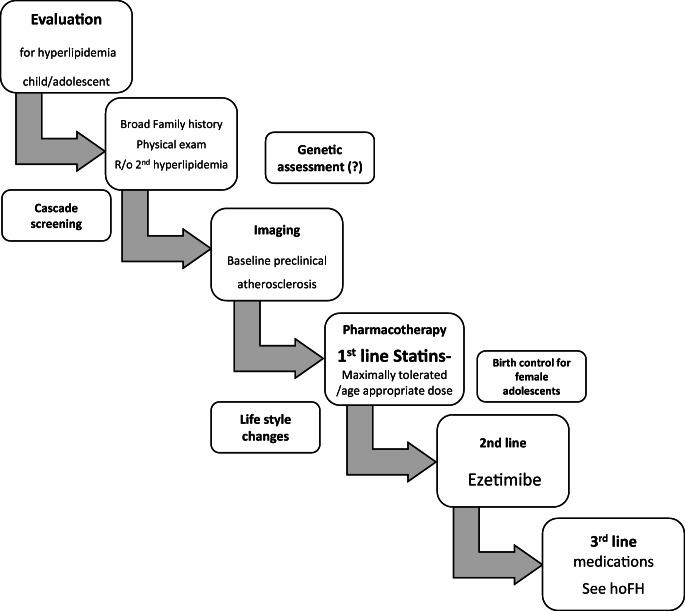
Treatment algorithm of heterozygous FH children and adolescents

Current heterozygous FH treatment options are also reported in Fig. 1.

## Life Style Changes

The role of a healthy lifestyle in children and adolescents including a heart-healthy diet, regular exercise, and tobacco abstinence should not be undermined as it has a positive impact, though small, on both plasma lipid levels and preclinical atherosclerotic changes [16].

## Clinical Management

The rational of early intervention in HeFH derives from the association of FH with elevated risk of premature accelerated ASCVD and that intensive LDL-C lowering treatment would halt or prevent vascular changes [1, 11, 17].

## Statins

Statins, 3-hydroxy-3-methylglutaryl-CoA reductase inhibitors, inhibit the hepatic cholesterol synthesis and increase the uptake of the LDL particles by the LDLR. There is abundant data in adults that statins confer cardiovascular protection both in primary and secondary prevention and reduce cardiovascular morbidity and mortality [18]. Statins are correspondingly the preferred pharmacologic therapy for FH in children and adolescents [15, 19, 20].

Several studies evaluated the short-term and long-term efficacy and safety of statin use in children and adolescents. In a study in which a 10-year follow-up was achieved in the majority of statin-treated HeFH children, no differences in growth, maturation, or educational level were noted, and long-term statin treatment initiated during childhood was associated with normalization of carotid intima-media thickness (CIMT) progression [21].

A recent comprehensive review assessed the currently available data regarding efficacy and safety of statin therapy in FH children. The review included nine randomized placebo-controlled studies with over 1000 HeFH pediatric patients. In terms of efficacy, statins reduced LDL-C levels on average by 32.15% at the end of follow-up, in the studies assessed, without significant effect on growth and maturation or any significant adverse events. The authors conclude that large, long-term randomized controlled trials are needed to establish the long-term safety issues of statins [22].

## Female Adolescents

Statin use is contraindicated in pregnancy and lactation; therefore, early statin initiation is of extreme importance in young girls and adolescents as they are more likely to experience interruptions in therapy during their lifetime. Suitable contraception is necessary to all post-pubertal young girls on lipid-lowering treatment (LLT) [23].

## Second-Line Lipid-Lowering Therapies

While most children respond well to statins and reach the desired LDL-C treatment goals [24], some of HeFH pediatric patients require a second line of therapeutic agents to achieve the LDL-C goal. Out of the additional agents that are available, niacin, bile acid sequestrants, and derivatives of fibric acid are seldom in use due to side effects and insufficient data in children. Ezetimibe, an intestinal cholesterol absorption inhibitor, is most used as a second-line agent [25]. Ezetimibe has a favorable side effect profile, reduces LDL-C levels, and decreases the rate of cardiovascular events in high-risk adult patients in the IMPROVE-IT trial [26]. In our practice, about 25% of children require the administration of ezetimibe in addition to a maximally tolerated dose of a statin. Ezetimibe is safe and well tolerated (Hofit Cohen et al. Unpublished data). Data regarding the use of ezetimibe in children is limited. A short-term trial of ezetimibe monotherapy with ezetimibe in mainly HeFH children 6–10 years of age resulted in significantly larger reductions of LDL-C (−27%) and TC (−21%) levels and APOB (−20%) vs placebo [27]. In another trial of adolescent HeFH patients, long-term co-administration of simvastatin with ezetimibe led to a greater reductions of LDL-C levels (−49.5%) than simvastatin monotherapy (−34.4%; *P* < 0.01) [28]. A recent study [29] analyzed the efficacy and safety of long-term co-administration of ezetimibe and statins in children and adolescents with HeFH. There were no clinically significant alterations in liver- or muscle-related laboratory tests or adverse effect on growth and maturation. Treatment goal of LDL-C (<135 mg/dL; 3.5 mmol/L) was achieved in more than two-thirds of the children.

## Third Line and Additional Lipid Lowering Agents

In severe dyslipidemia, third-line agents may be considered—such as PCSK9 inhibitors. In a recently published paper, evolocumab, a monoclonal antibody directed against PCSK9 with powerful lipid lowering effect, was given in pediatric patients with HeFH. The HAUSER-RCT trial was a 24-week, randomized, double blind, placebo-controlled trial that evaluated the efficacy and safety of evolocumab in that population. Following 24 weeks of treatment, the mean change in LDL-C levels was −44.5% in the evolocumab group and − 6.2% in the placebo group, (*P* < 0.001), with no difference in the incidence of adverse events between the evolocumab and placebo groups [30••]. Additional investigational therapeutic agents are lomitapide, mipomersen, inclisiran, and evinacumab.

## Evaluation of Preclinical Atherosclerosis

Non-invasive cardiac and vascular imaging techniques are used to evaluate the extent of atherosclerotic vascular changes in children and adolescents and assess the impact of LLT. Abnormal imaging findings were reported in young HeFH patients, with increased CIMT, and abnormal flow-mediated dilation (FMD) [31, 32]. Several studies have demonstrated improvements in these preclinical markers of atherosclerosis.

## Carotid Intima Media Thickness

Wiegman et al. demonstrated that 2 years of pravastatin therapy induced a significant regression of CIMT compared to placebo in children aged 8 to 18 years with HeFH [33]. After the original study, in a follow-up study, the patients in the placebo arm were switched to pravastatin, and CIMT measurements were performed following on average, four and a half years of treatment. The results showed that the age in which statin therapy was started was an independent predictor for CIMT after follow-up and that timely initiation of statin treatment at a young age delayed the progression of CIMT in adolescents and young adults [34]. Braamskamp et al. evaluated the effect of 2-year treatment with rosuvastatin on CIMT in HeFH children (age 6 - < 18 years). At baseline measurements, CIMT was considerably higher for the HeFH children compared with the unaffected siblings (*P* = 0.001). Throughout the follow-up, the change in CIMT was 0.0054 mm per year in HeFH children and 0.0143 mm per year in the healthy siblings (*P* = 0.002). The authors concluded that rosuvastatin treatment for 2 years resulted in significantly less progression of the increased CIMT in HeFH children compared with their unaffected siblings. No difference was found in the CIMT at the end-of-study evaluation, supporting the concept of early initiation of statin treatment in children with HeFH [31].

## Flow-Mediated Dilation

De Jongh demonstrated a positive change of the endothelial function as measured by FMD in a study of HeFH patients. A group of HeFH children and adolescents, 9 to 18 years old, treated with simvastatin was compared with healthy, non-FH controls and HeFH patients treated with placebo. At baseline, FMD was impaired in children with FH versus non-FH controls (*P* < 0.024) and was significantly improved in the statin HeFH group remaining stable without change in the placebo HeFH group at 28 weeks of treatment [32].

## Cardiac Computed Tomography (CT) and Calcium Score

Coronary artery calcium (CAC) score is a marker of subclinical atherosclerosis burden. As the clinical course of ASCVD in subjects with HeFH is heterogeneous, CAC score has the potential to improve risk stratification in HeFH patients. It has been evaluated in FH patients over the age of 18 and was independently associated with ASCVD events. Cardiac CT and CAC score may help to further stratify near-term risk in patients who may need additional LLT. As of 2020, there is no data regarding the pediatric age group [35, 36].

## Atherosclerotic Cardiovascular Disease Outcome of Early Treatment

Currently, a universal consensus has been established in support of early preventive measures in FH patients. The European Atherosclerosis Society (EAS) consensus panel and the American College of Cardiology–American Heart Association guidelines both support initiation of statin therapy from the ages of 8–10 years [15, 16]. The lipid-lowering effect of statin therapy is well recognized in both children and adults; however, long-term follow-up data focusing on cardiovascular outcomes in treated children is lacking. While in adults with FH, the importance of statin treatment in the prevention of cardiovascular disease was recalled in several long-term studies [37, 38]. With statin treatment, HeFH patients had highly significant reduction of CHD morbidity and mortality both in primary prevention and secondary prevention [38]. Luirink et al. [39•] reported a 20-year follow-up study in children with HeFH who started statin treatment in childhood. The patients, with genetically confirmed FH, took part in a previous trial of 2-year treatment with pravastatin and were followed up, alongside their unaffected healthy siblings. Additionally, progression of subclinical atherosclerosis (measured by CIMT) was assessed in the HeFH patients and their siblings, and the incidence of clinical cardiovascular disease among the HeFH patients was compared with that of their affected parents. The mean LDL-C level in the patients decreased by −32% from the baseline level. Mean progression of CIMT was the same over the follow-up period in the treated HeFH patients and their siblings. The cumulative incidence of cardiovascular events and death from cardiovascular causes at 39 years of age was lower among the patients with FH than among their affected parents, who started statin treatment at a later age (1% vs 26% and 0% vs 7%, respectively). The prolonged follow-up study has demonstrated that initiation of statin therapy during childhood in patients with FH was safe and effective, and above all, it significantly slowed the progression of CIMT and reduced the risk of ASCVD in adulthood [39•].

## Homozygous Familial Hypercholesterolemia

Homozygous familial hypercholesterolemia (HoFH) is a genetic disease due to a mutation inherited from both parents. In most cases, the mutation is in the LDLR gene. HoFH causes high LDL-C levels, increasing from an early age. Subjects with mutation in their genes leading to HoFH are simple homozygotes (same mutation within the same gene on each allele), compound heterozygotes (different mutation within the same gene on each allele), and double heterozygotes mutations on 2 different genes). Furthermore, HoFH may also be inherited recessively (homozygous LDLR activating protein—LDLRAP 1/autosomal recessive hypercholesterolemia or ARH). Further bi-allelic mutations may occur on genes encoding for APOB and PCSK9. HoFH is a rare disease, with an estimated historic prevalence of 1/1,000,000 population [2]. However, more recent work reported that the incidence is much higher and close to 1:250,000 [7, 40]. High levels of LDL-C due to the diminution in LDLR activity can lead to very elevated LDL-C levels from birth, accelerated narrowing and hardening of the arteries (atherosclerosis), and premature death from myocardial infarction/acute coronary insufficiency, also frequently associated with valvular and supravalvular disruptive atheroma of the aortic root [41, 42]. In HoFH, the clinical features of the disorder include tendon and skin xanthomatas, corneal arch, and a TC level > 600 mg/dL. Keeping cholesterol in a healthy range is important for everyone, but especially for patients with HoFH. In fact, patients with HoFH have difficulty in achieving the correct LDL-C levels with conventional medical treatment, such as diet and usual lipid-lowering drugs. If untreated the average age of death is 18 years, although deaths before the age of 5 years were reported. Due to the relative rarity of HoFH and understandable ethical and clinical priorities there have been no randomized clinical trials on usual and novel LLT. However, a remarkable bulk of evidence suggest that an early, intensive, and customized multimodal treatment approach with drugs and LA is beneficial [43–46].

## Management of Homozygous FH

Statin therapy is often inadequate to achieve required treatment goal, despite the use of maximal doses and/or combination therapies with ezetimibe, resins sequestering bile acids, or fibrates [47, 48]. Therefore, novel therapeutic strategies in addition to statin therapy are necessary. Four classes of new effective agents decreasing LDL-C are currently developed or in the process of advanced human experimentation. Further studies on long-term safety and efficacy, along with tolerability over time, are necessary. A first line of development includes direct monoclonal antibodies with PCSK9, which reduce LDL-C up to 60–70% in FH heterozygous subjects treated with statins. Most LDL receptors are reused (re-cycling), and the PCSK9, complexed to the LDLR, prevents its intracellular recycling, favoring its degradation and thus reducing the number of receptors present on the cell membrane. The block with a monoclonal antibody of PCSK9 promotes the recycling of LDLRs on the surface of the cell membrane where they are more numerous, inducing the uptake of LDL from the circulation and the reduction of their plasma levels [49–51]. PCSK9 inhibitors in combination with LA allow for more effective treatment in a considerable percentage of subjects with severe FH. However, HoFH patients due to receptor negative, or *null* mutations, remain impossible to be adequately pharmacologically treated without LA [52]. The impact of alirocumab on cardiovascular outcomes in HoFH will need to be evaluated in future studies [53]. A further attempted approach consists in the use of the anti-sense oligonucleotide mipomersen, which works by reducing the hepatic production of APOB through the degradation of mRNA, resulting in a reduction in assembly and the production of all atherogenic lipoproteins. In FH heterozygous subjects with CHD on maximum tolerated dose of statins, mipomersen reduced LDL-C by −28%, lipoprotein (a) [Lp (a)] by −21%, and APOB of −26%. Side effects are frequent and include reactions to the injection site and flu-like symptoms [54]. Lomitapide inhibits the microsomal transfer protein of triglycerides (MTP), interfering in hepatic assembly and secretion of very-low-density lipoprotein (VLDL), since this protein is critical for transfer of triglycerides (TG) on APOB. In subjects with homozygous FH treated by diet only, the maximum reduction obtained with lomitapide was −51% for LDL-C, −79% for VLDL-C, −65% for TG, − 56% for APOB, and − 15% for Lp (a). However, fatty liver and gastrointestinal side effects occur frequently [55]. Cholesterol ester transfer protein inhibitors (CETP) (anacetrapib and evacetrapib) reduce transfer of cholesterol esters from high-density lipoprotein (HDL) to VLDL and LDL and the transfer of TG from the latter to HDL. Anacetrapib lowers the LDL-C and Lp (a) up to 40% when added to statin treatment in subjects without FH and increases HDL-C levels by 140% [56]. Gene-silencing therapies against PCSK9 (inclisiran) and based on the inhibition of angiopoietin-like 3 (ANGPTL3) (evinacumab) are under advanced clinical experimental evaluation. Evinacumab, a monoclonal antibody against ANGPTL3, has shown potential benefit in adult patients with HoFH. In HoFH patients receiving maximum doses of lipid-lowering drugs, the reduction from baseline in the LDL-C levels in the evinacumab group, as compared with the placebo group, resulted in a between-group difference of 49.0% at 24 weeks [57, 58]. Patients with HoFH are difficult to treat, especially in those with limited residual activity of the LDLR. Therefore, to reduce the risk of developing premature ASCVD, novel therapeutic agents are needed to lower LDL-C levels. This is particularly important for the adolescent population with HoFH. The impact of evinacumab on the subgroup of adolescents will be evaluated as the study on adolescents is still ongoing. According HEART UK, the risk of early use of lipid-lowering drugs beyond their licensed age restriction may be reasonably acceptable against the risk represented by invasive procedure such as liver transplantation or other ineffective treatment in HoFH patients. The recommendation is that HoFH children should be treated early by a specialist with expertise in the management of HoFH. LA should be initiated as soon as possible, but it is often difficult to get children younger than 5 to accept the extracorporeal procedure. It should be considered early and started even before the age of 7. LA combined with statin and ezetimibe should also be considered. Evolocumab can be considered from the age of 12 if treatment targets are not achieved [59]. More in general, earlier treatment can be considered if evidence of atherosclerosis progression is documented. Coronary angiography, performed by a pediatric cardiologist with expertise in HoFH, is preferred to stress testing in severely affected children in general clinically asymptomatic. Prescription of lipid-lowering drugs beyond license should be considered in the context of a multidisciplinary team and only after approval by local ethic committee. Risk may be balanced against the benefit of avoiding aortic arch surgery or the risk of liver transplantation, which might be considered as a last resort if there has been suboptimal achievement of therapeutic target lipid and lipoprotein levels in HoFH children [59]. Rosuvastatin treatment is currently prescribed at the diagnosis, but statins, in general, have not been formally evaluated/approved for HoFH children. The HYDRA study was a randomized, placebo-controlled trial of any statin in pediatric HoFH patients aged 7 and demonstrated significant reduction in LDL-C with rosuvastatin 20 mg daily of 85.4 mg/dL (−22.3%) compared with placebo. Rosuvastatin 20 mg daily was reasonably well tolerated. The largest mean reduction in LDL-C levels with rosuvastatin was observed in the subgroup with the most residual LDLR activity. The HYDRA study demonstrated effective LDL-C reduction with rosuvastatin 20 mg daily alone or in combination with ezetimibe and/or LA and resulted in the US Food and Drug Administration approval of rosuvastatin for the treatment of HoFH children and adolescents 7 to 17 years of age [60]. Lomitapide is not approved for use in children but has been made available through an expanded access program or on a named patient basis, after approval of local ethic committee. Ben-Omran et al. reported a case series of 11 HoFH patients in 10 different centers, younger than 18 years of age (mean 11.6 ± 1.1 years, 64% males), with signs of ASCVD, who have been given lomitapide (mean dose 24.5 ± 4.3 mg/day; mean exposure 20.0 ± 2.9 months). Lomitapide was uptitrated from 2.5 mg or 5 mg/day. In the 11 HoFH children, mean baseline LDL-C was 419 ± 74.6 mg/dL reduced by lomitapide to a nadir of 176.7 ± 46.3 mg/dL (−58.4 ± 6.8% decrease). Six patients achieved recommended target levels for HoFH children below 135 mg/dL, and five had LA frequency reduced. Adverse events were usually gastrointestinal, occurred early in the treatment course. Three patients showed increases in liver function tests. For two patients, lomitapide was downtitrated from the initially given doses [61].

## Lipoprotein Apheresis

**Fig. 2:**
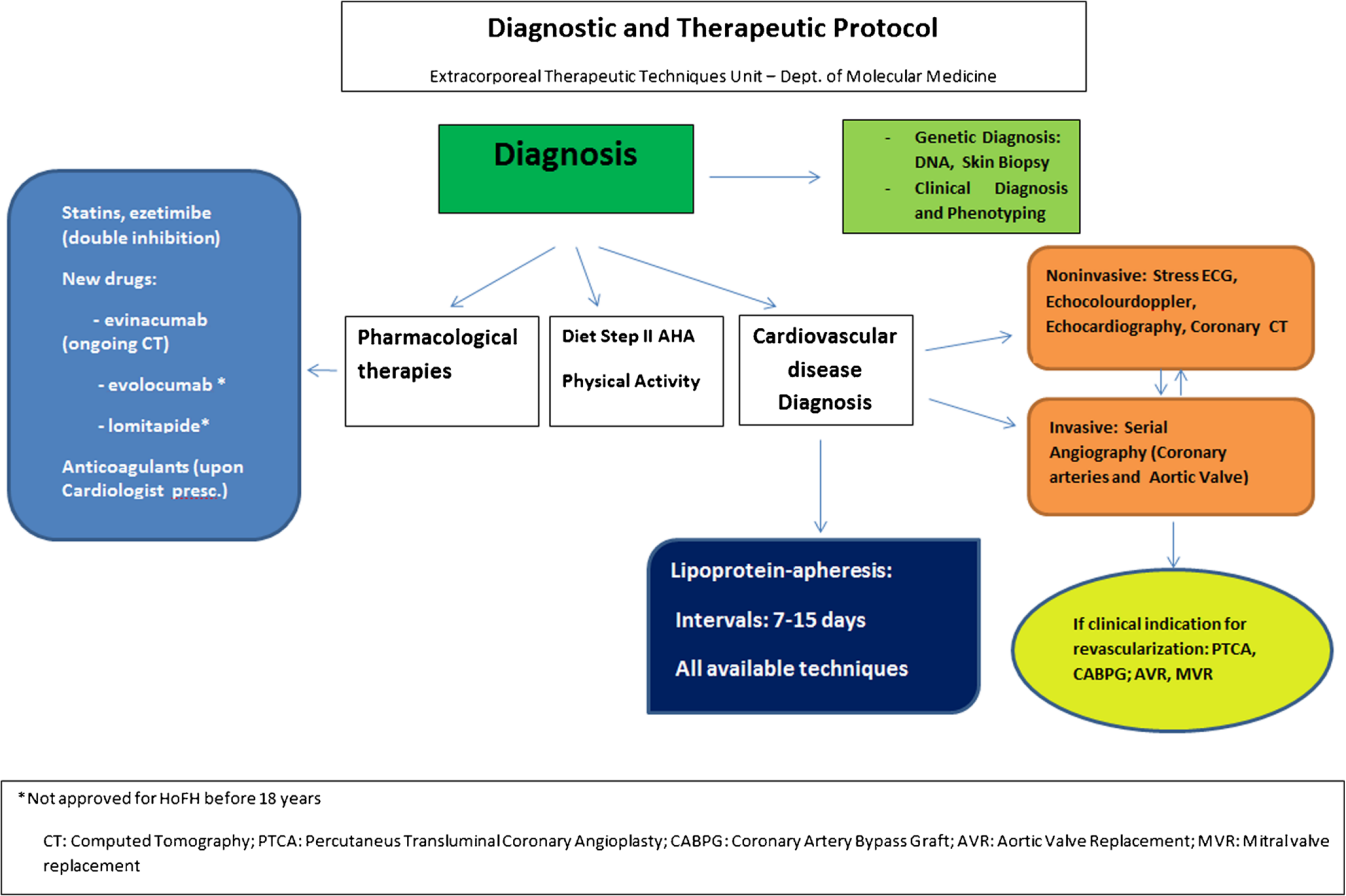
Management of homozygous FH children and adolescents. Diagnosis and treatment protocol in Rome

In individuals at very high cardiovascular risk FH and/or with CHD, with high LDL-C levels despite therapy, or who showed intolerance to statins, treatment with LA should be considered. Weekly or bi-weekly apheresis reduces LDL-C by 50–70% and has clinical benefit in subjects with severe FH [48, 62, 63]. Interestingly, very young HoFH children and adolescents have been treated by LA during the last 2 decades. In 1997 Stefanutti et al. reported the first historical treatment experience by dextran sulfate cellulose LA (known as LDL apheresis at that time) of a 4.5-year-old HoFH girl with coronary artery disease (CAD). The young girl tolerated LA without any clinically significant complication [64]. The case report suggested the possibility of early beginning of extracorporeal treatment with LA in HoFH children. Accordingly, in 2001 the same group successfully treated a 3.5-year-old girl. The girl was the youngest patient ever treated with LA, and even in this case, no significant side effects have been observed [65]. LA proved to be a safe and effective therapeutic procedure, and these pivotal experiences have stimulated other authors worldwide to start LA in very young children. Data from a registry of 29 patients submitted to LA before 18 years at 15 sites during the 11 years since the approval of LA by the US Food and Drug Administration were analyzed [66]. The youngest patient was 3-year-old at the start of apheresis. Hudgins et al. reported that LA was well tolerated by pediatric HoFH patients [66]. In 2008 Palcoux et al. concluded that LA can be recommended for the treatment of HoFH, even in young children. LA showed effectiveness on biological parameters and skin lesions preventing cardiovascular events [67]. Further clinical experience on a 4.5-year-old HoFH child was reported by Lefort [68]. Coker et al. in 2009 reported their findings on two young patients who were part of a cohort of ten patients treated with LA. The adverse events occurred in their cohort are like those reported in adults [69] (Table 1). The HoFH children submitted to LA in Rome by Stefanutti C et al. were first managed according a customized diagnostic algorithm which provides a very careful preliminary assessment of coronary arteries, aortic valve, and cardiovascular profile by means of non-invasive examinations to invasive coronary angiography. This clinical management protocol was recently adapted and updated introducing novel available cardiovascular imaging techniques but remained substantially unchanged over the years (Fig. 2). Thresholds for initiating the procedure of LA vary from country to country. For example, in Germany FH subjects with CHD suitable for LA are those with LDL-C levels greater than 2.6 mmol/L (100 mg/dL) notwithstanding drug therapy, while in the USA, the corresponding threshold is 5.2 mmol/L (200 mg/dL). LA combined with high-dose statin and ezetimibe can slow the progression of atherosclerosis. Adjunctive use of novel compounds such as lomitapide, evolocumab, and, probably in future, inclisiran and evinacumab could facilitate the attempt of achieving the goal of interrupting atherosclerosis progression by enhancing the reduction in LDL-C. The bulk of available evidence converges on the need for a customization of the treatment of HoFH using increasingly effective drugs to reduce atherogenic lipoprotein levels in association, where possible, with LA. This explains, at least in part, the outcomes of the “Sino-Roman Study,” a cross-national investigation on cardiovascular survival in HoFH. This retrospective multinational, multicenter clinical study was undertaken comparing CVD-free survival and mortality in 44 HoFH patients who were treated with statins but not LA, from a center in Beijing, China, and 18 HoFH patients who were treated with LA and novel therapies from an early age, from a center in Rome, Italy. CVD-free survival and survival were significantly reduced in Italian patients compared with the Chinese patients after 30 years of follow-up (log-rank *P* < .01). In a pooled analysis, cardiovascular survival was significantly increased with earlier age at treatment, longer duration of treatment, and lower on-treatment LDL-C concentrations (*P* < .05). In addition, the probability of a CVD event and death were increased in patients that carried a null mutation in the LDLR or had elevated Lp (a). The authors concluded that CAD outcomes in patients with HoFH can be significantly improved with earlier and marked LDL-C lowering with usual and innovative drugs in combination with LA. This has major implications for countries, such as China, where the models of care for HoFH remains underdeveloped [46]. LA is a safe well-tolerated outpatient treatment to lower LDL-C and Lipoprotein (a) by 60–70% and is currently the only effective, safe, and convincing therapeutic option in patients with elevated Lp (a) and progressive CVD. Major therapeutic effect of LA is preventing cardiovascular events [48]. The Pro (a) LiFe study investigated with a prospective multicenter design the long-term preventive effect of LA on incidence rates of cardiovascular events prospectively over a period of 5 years in 170 consecutive patients who were submitted to regular LA. During a median period of 4.7 years of the pre-LA period, Lp (a)-associated progressive CVD became apparent. One hundred fifty-four patients (90.6%) completed 5 years follow-up. Significant decline of the mean annual major adverse cardiac event (MACE) rate was observed from 0.41 ± 0.45 2 years prior to regular LA to 0.06 ± 0.11 during 5 years with regular LA (*P* < 0.0001). Results of 5 years prospective follow-up confirmed that LA exerts a major therapeutic effect in preventing cardiovascular events in patients with elevated Lp (a) associated with progressive CVD [70]. Klaus G et al. (2018) reported a study on seventeen patients genetically diagnosed as HoFH or compound HeFH submitted to LA before the age of 18 enrolled in an open, observational, retrospective multicenter study, involving ten specialized nephrological centers throughout Germany. Interestingly, five patients were submitted to apheresis when they had a body weight ≤ 20 Kg. Patients mean age at diagnosis was 5.8 years ±2.5 years. All patients had elevated LDL-C levels prior to LLT with a mean LDL-C level of 19.6 mmol/L ± 5.3 mmol/L (756 mg/dL ± 206 mg/dL). All had a positive family history for CVD. Seven patients within this cohort were siblings from three parents. All patients had documented mutations with complete or partial impairment of LDLR function as the underlying genetic cause of FH. Thirteen patients (76%) remained clinically stable with no cardiovascular events, no need of revascularization, and no progression of ASCVD. ASCVD progression occurred in four patients with cardiovascular events (24%) including one patient who died because of progressive coronary and cerebrovascular diseases. Patients were given statins, ezetimibe, or both. The retrospective analysis showed that further standardization of LLT, initial, or subsequent choice of a high-intensity statin, early combination with ezetimibe, PCSK9 antibody, produced additional reduction of LDL-C, assuming full tolerability of drugs. Mean age at first LA was 7.8 years ±2.8 years. Multimodal LLT including chronic LA resulted in a 73% (± 9.1%) mean reduction of LDL-C levels compared to untreated patients. Regular LA contributed to a 62% reduction from untreated baseline LDL-C > 600 mg/dL. The authors suggested that this outcome represents per se a remarkable reduction of cardiovascular risk in young HoFH patients. Furthermore, in three patients, the above-mentioned multimodal therapeutic approach including drugs and LA resulted in mean LDL-C levels below the pediatric target of 3.5 mmol/L (135 mg/dL). In two patients, LA frequency was twice per week. Increase of LA frequency, e.g., twice per week, is an option to further decrease mean LDL-C and was shown to be more effective compared to the increase of treated plasma volume. However, weekly, or biweekly LA treatment still represents a standard of care in several European countries. The authors suggest that quality of life including psychosocial aspects, e.g., treatment-related stress, distance between patients’ homes and the treatment center, and impairment of patients and parents’ daily routines should also be considered. Interestingly, the authors concluded that their findings confirm that LA is a remarkable component of multimodal LLT still indispensable for HoFH and compound HeFH cases to effectively lower LDL-C levels and is safe in very young FH children. Implementation of a standardized multimodal strategy of LLT escalation is desirable and should be investigated to optimize outcomes. LA is a complex treatment for patients and their families, indeed. However, in our knowledge, LA was not associated with impairment of health-related quality of life [71]. In a very recent study (2020), the authors set a database on therapeutic characteristics and outcomes of a group of HoFH patients who have been submitted to LA during their childhood. The study was designed as an observational international multicenter cohort study. The web-based database was opened for patient-data registry in 2016. The data reported were entered into the online data server up until November 2018. The authors reported that in their cohort, LA was initiated in 47% of patients after the age of 8, and the median time between diagnosis and starting LA was 2.8 (1.0–4.7) years. The fact that none of the patients had experienced CVD around the time of diagnosis and six (12%) developed major CVD between diagnosis and LA underlines the importance of starting treatment at the earliest possible age, according Luirink K et al. The authors found also substantial different approaches with respect to vascular access for LA treatment. The differences were associated with the medical specialty of the physician in charge. Cardiologists never applied an arteriovenous (AV) shunt for LA treatment in children in contrast to pediatric nephrologists who predominantly used AV shunts. This preference of (pediatric) nephrologists might be related to their familiarity with extracorporeal procedures applied to other severe pathologies. An AV shunt makes vascular access relatively easier but not less traumatic for children. It also enables physicians to increase the blood-flow and reduce treatment time. However, AV shunts are known to produce a wide range of serious side effects leading even to severe adverse events [72•]. A possible explanation for the concern for AV shunt use among cardiologists is the associated increased cardiac output and dilatation of all cardiac chambers [73, 74]. The cardiac burden of the increased circulating volume induced by an AV shunt may provide a cardiac risk on long-term. So far, this association has only been described in renal patients who suffer from CVD burden for other reasons. Long-term follow-up of the cardiac burden related to the use of AV shunts in children is necessary and mandatory. The authors’ findings confirmed that LA is a safe treatment with rare side effects inducing significant reduction of LDL-C in HoFH children. Moreover, Luirink et al. found significant short-term effects of LA with respect to disappearance of xanthomatas in a linear relationship with the duration of LA treatment. LDL-C reduction over 70% per extracorporeal procedure can be achieved in HoFH children. However, despite the potential efficacy of LA, only a small number of patients reached the recommended mean LDL-C over time. These data suggest that treatment must be optimized and better customized. The average LDL-C can be lowered by increasing the frequency of LA to at least once per week and/or increasing the plasma volume processed. Long-term follow-up data on the effect on ASCVD or surrogate markers for ASCVD in combination with quality-of-life assessment must provide evidence for the need of further optimization/customization of LA treatment [72].

**Table 1:** Children with homozygous FH submitted to lipoprotein apheresis before age 5

Source	Age (yrs)	LDL-C (mmol/l)	Method*	Frequency (days)	Duration (month/yr)
Stefanutti (1997)	4.5	19.4	DSA	15	1.5 m
Stefanutti (2001)	3.5	24.3	DSA	15	4.5 m
Hudgins (2008)	3	18.4	DSA	14	32 m
Palcoux (2008)	3.5	23.0	DSA/DALI	14	7.7y
	4.8	19.2	DSA/DALI	14	21y
Lefort (2009)	4.5	24.9	DALI	14	12 m
Coker (2009)	4	20.9	DFPP/APP	14	22 m
	2	18.3	DFPP/APP	14	58 m
Mann (2013)	2.25	23.2	PP/DSA	7	72 m
Mean	3.6	21.3		13	1.5 m–21y

*DSA dextran suplhate adsorption, *DALI* direct adsorption of lipoproteins, *DFPP* double filtration plasmapheresis, *APP* adsorption plasmapheresis, *PP* plasmapheresis

## Practical Recommendations for the Diagnosis and Treatment of Children and Adolescents with FH

The most important approach to detect children with FH is that both the general practitioner (GP) as well as the pediatrician ask during every routine visit for CVD and/or hypercholesterolemia in the family. Usually this has to be done repeatedly, because not all parents are aware of the pathology. The second way is either the so-called cascade screening (to test all family members starting from the proband, who has been diagnosed to be affected with hypercholesterolemia) or, otherwise, the so-called child-parent screening, if hypercholesterolemia is known affecting a child. In this regard there are reports from programs held during vaccination visits; the ideal procedure would be to screen all children before the age of approximately 10 years. However, it has been shown that the discrimination between affected and non-affected children is ideal before the age of 10 years. The approach is different if the child is diagnosed as HoFH and the clinical management is rather different and must be anticipated.

## Diagnosis

1. If the LDL-C level in the child exceeds the 95th percentile for age and sex (in Europe usually130–135 mg/dL) along with a positive familial history, the diagnosis of HeFH is very likely.
2. In addition, a genetic test for DNA mutation can be performed; however, this is not everywhere possible and also expensive and is usually done in homozygous rather than heterozygous FH.
3. The next step is to inform carefully the families about the later risk of the genetic disorder and the chance to prevent or even to postpone CVD in early adulthood. This process is very important, because many parents hesitate to start a treatment of children without symptoms.
4. In children with LDL levels over 190 mg/dL or a substantial family history of premature CVD, it has been suggested to begin the treatment at the age of 6 years.
5. In several western countries, CIMT testing in almost all children as a noninvasive mean to assess pre-clinical vascular changes and repeated as follow-up in 1–2-year intervals (at the same center possibly) can be clinically meaningful;

## Treatment

1. If the parents are well informed and are willing to be involved in a lifelong treatment—which is very likely to be successful—a discussion about healthy life style is essential. It has been shown that a diet (low fat and high content of monounsaturated and polyunsaturated fatty acids) is able to lower LDL-C by up to 15%, and a substitution of animal protein by soy protein can achieve a further LDL-C lowering effect of approximately 10% [75, 76].
2. This is noteworthy, that in some children with HeFH, the introduction of drug therapy can be postponed, because the LDL-C levels can be lowered by means of diet so that premature administration of drug therapy would not be justified or at least postponed [77].
3. Whereas after at least 3 months life style change LDL-C levels could not be lowered (<150–160 mg/dL), drug therapy is necessary or should be recommended. Statins are the drugs of choice, and several studies were able to show that they are safe and have a very low incidence of side effects. Many pediatricians use rosuvastatin for children with HeFH starting with a dose of 2.5 mg/day (Prof. Kurt Wildhalm, 2020, unpublished). Local differences are sometimes justified.
4. It would be possible to start the initial dose of a statin, after a trial of therapeutic life style changes with pravastatin 10 mg/day or simvastatin 10 mg/day or atorvastatin 10 mg/day or rosuvastatin 10 mg/day, and later increase the dose up to 40 mg/day according to the most appropriate clinical target.
5. Ezetimibe is usually added as a 2nd-line therapy and usually is very well tolerated alone or in combination with statins.
6. The question about which are the desirable LDL-C levels for FH children cannot be really answered so far, because long-term data on the effect of drug treatment are scarce. Lab tests including GOT, GPT, CK, and other routine parameters should be done approximately 3 times a year.
7. In homozygotes FH lipoprotein apheresis is recommended starting as soon as possible depending on body mass index, the vein access accessibility, the child’s behavior, and willingness to be submitted to LA along with the parents full collaboration and awareness;
8. Lomitapide, PCSK9 inhibitors, and evinacumab were used recently for compassionate purpose (no current approved indication of the above-mentioned novel lipid lowering drugs in both HeFH and HoFH children) or within the frame of ongoing clinical trials (data only partially published or unpublished).

## Conclusion

Dyslipidemias represent one of the most important causative factors of CVD. Dyslipidemias are clinical conditions in which qualitative and/or quantitative alterations of lipids and plasma lipoproteins are evincible. Dyslipidemias can be primary (genetic) or secondary to other pathology. Among the primitive forms, FH is the most frequent among the genetic causes of early CVD as induces lifelong exposure to high LDL-C levels. Millions of people around the world do not know they are along with their families, at high risk of a cardiac ischemic event (e.g., myocardial infarction), cerebral (stroke), or peripheral vascular (carotid atheromasia, peripheral arterial disease). If left untreated, FH men and women in the heterozygous form develop such pathologies respectively before 55 and 60 years, while young FH homozygotes children and adolescents not early submitted to adequate treatment die before the age of 20. Early diagnosis would allow adequate prevention, and appropriate treatment can reduce mortality and cardiovascular morbidity. Individuals with FH have elevated LDL-C levels from birth; atherosclerosis begins and develops in childhood, an evidence affecting even the prognosis. Therefore, to reduce the lifetime risk of CVD, FH patients should be diagnosed as early as possible and appropriate treatment initiated. Diagnosis of FH is often made utilizing clinical and biochemical criteria (Dutch Lipid Clinic Network or the Simon Broome Register Group). Genetic molecular evaluation is an additional strongly recommended option to confirm the diagnosis. The initial therapeutic approach necessitates a combination of nutritional and lifestyle changes from an early age with therapeutic intervention. Noninvasive evaluation to assess pre-clinical atherosclerosis must be performed (carotid IMT, study of endothelial function). Expert consensus recommends a target LDL-C levels of less than 3.5 mmol/L (130 mg/dL) from age 10 years, or 50% reduction from pre-treatment levels for children 8–10 years. Long-term, continuous treatment with LA can mobilize a significant amount of cholesteryl esters from intracellular storage, and weekly or biweekly LA results in regression of xanthomatas and xanthelasmas in young individuals with severe genetic dyslipidemia. Clinical evidence also suggests that long-term LA contributes to plaque regression and/or stabilization, as well as improvements in prognosis. In HoFH, marked lowering of LDL by LA appears to improve coronary atherosclerosis and aortic valvular disease and increase longevity, particularly when treatment is initiated at an early age. Initiation of LA in very young, low body mass index children can be very challenging but is routinely achieved by skilled medical teams. Although many important advances are being made in the field of lipid-lowering therapy, many dyslipidemic patients still do not attain sufficient lipid lowering, and, as a result, they remain at high CVD risk. Novel lipid-lowering agents may be promising and may have the potential of further reducing CVD risk if conventional therapies do not yield adequate results. Novel lipid-lowering drugs may yield in future better results when combined with LA or given without LA if this facility is not available in a customized, careful, and appropriate multimodal treatment approach [48, 78].

## Abbreviations

ASCVD: Atherosclerotic cardiovascular disease
APOB: Apolipoprotein B
CHD: Coronary heart disease
CAD: Coronary artery disease
FH: Familial hypercholesterolemia
HeFH: Heterozygous familial hypercholesterolemia
HoFH: Homozygous familial hypercholesterolemia
TC: Total cholesterol
LDL: Low-density lipoprotein
LDL-C: Low-density lipoprotein cholesterol
Lp (a): Lipoprotein (a)
LDLR: LDL receptor
VLDL: Very-low-density lipoprotein
TG: Triglycerides
HDL: High-density lipoprotein
PCSK9: Proprotein convertase subtilisin/kexin type 9
IMT: Intima-media thickness
CIMT: Carotid intima-media thickness
FMD: Flow-mediated dilation of the brachial artery
CAC: Coronary artery calcium (score)
CT: Cardiac computed tomography
LDLRAP1: Homozygous LDLR activating protein
ARH: Autosomal recessive hypercholesterolemia
MTP: Microsomal transfer protein (of triglycerides)
ANGPTL3: Angiopoietin-like 3
CETP: Cholesterol ester transfer protein
LLT: Lipid-lowering therapy
LA: Lipoprotein apheresis
MACE: Major adverse cardiac event
